# Abstract of Meteorological Observations for October, 1855, Made at Philadelphia, Pa.

**Published:** 1855-12

**Authors:** James A. Kirkpatrick


*Abstract of Meteorological Observations for October, 1855, made at Philadelphia, Pa. Latitude* 39° 57′ 28″ *N., Longitude* 75° 10′ 40″ *W. from Greenwich.* By PROF. JAMES A. KIRKPATRICK.

| 1855. October. | Barometer. Daily Mean | Barometer. Mean Daily Range. | Thermom. Daily Mean | Thermom. Mean Daily Range | Dew Point 2 P.M. | Force of Vapor 2 P.M. | Rel. Humid. 2 P.M. | Rain. | Prevailing Winds. | Remarks. |
|---|---|---|---|---|---|---|---|---|---|---|
| | Inches. | Inches. | Deg. | Deg. | Deg. | Inches. | Hunds. | Inch. | Points. | |
| 1 | 29.651 | .265 | 72.2 | 6.2 | 65.5 | .628 | .72 | | NW. | M. and aft. cl'dy; ev. clear. |
| 2 | 29.545 | .106 | 68.2 | 4.0 | 61.8 | .551 | .75 | 0.326 | (Var.) | Cloudy; m. and ev. *rain*. |
| 3 | 29.491 | .100 | 61.7 | 6.5 | 57.3 | .470 | .73 | 0.072 | W. | M. and ev. clear; aft. cl'dy; n't *rain*. *Bar. l'st* 29.458. |
| 4 | 29.646 | .155 | 61.0 | 1.3 | 50.1 | .362 | .55 | | WNW. | M. cl'dy; aft. and ev. cl'r; 7 to 11 P.M. brilliant *aurora*. |
| 5 | 29.761 | .116 | 69.0 | 8.0 | 56.5 | .457 | .49 | | SW. | Clear. *Therm. highest* 77° |
| 6 | 29.673 | .088 | 64.8 | 5.5 | 68.5 | .701 | .92 | 0.683 | S. | Cloudy; m. and aft. *rain*. |
| 7 | 29.898 | .225 | 51.0 | 13.8 | 36.5 | .216 | .43 | | WNW. | M. and ev. clear; aft. cl'dy; first *hoar frost* observed. |
| 8 | 29.928 | .045 | 51.8 | 2.7 | 34.9 | .203 | .42 | 0.005 | WSW. | M. clear; aft. cloudy; ev. drizzling *rain*. |
| 9 | 29.994 | .066 | 55.7 | 4.0 | 48.6 | .343 | .58 | | (Var.) | M. cl'dy, aft. and ev. clear. |
| 10 | 30.046 | .059 | 62.0 | 6.3 | 58.0 | .482 | .66 | | SW. | M. hazy, aft. and ev. clear. |
| 11 | 29.851 | .195 | 66.0 | 4.0 | 55.2 | .436 | .48 | 0.467 | SE. | M. fog; aft. cl'r; ev. & n't r'n. |
| 12 | 29.806 | .080 | 49.7 | 16.3 | 40.9 | .256 | .62 | | NW. | M. & aft. cloudy; ev. clear. |
| 13 | 29.820 | .014 | 42.2 | 7.5 | 37.4 | .224 | .79 | 0.038 | WSW. | Cl'dy; m. & night drizzling |
| 14 | 29.822 | .017 | 49.7 | 7.5 | 39.6 | .243 | .57 | | SW. | Cloudy. [*rain*. |
| 15 | 29.853 | .031 | 55.2 | 5.5 | 42.4 | .271 | .47 | | SW. | M. hazy; aft. and ev. clear. |
| 16 | 29.977 | .125 | 54.3 | 3.8 | 46.1 | .312 | .56 | | NW. | M. hazy; aft. and ev. clear. |
| 17 | 30.038 | .074 | 48.7 | 5.7 | 40.2 | .249 | .54 | | WNW. | Clear. |
| 18 | 29.971 | .067 | 54.0 | 5.3 | 50.8 | .372 | .63 | | SW. | M. hazy, aft. and ev. clear. |
| 19 | 29.951 | .022 | 59.8 | 5.8 | 60.2 | .522 | .79 | | SW. | M. fog, aft. and ev. clear. |
| 20 | 29.821 | .130 | 59.8 | 4.0 | 60.6 | .530 | .97 | 0.491 | NNE. | M. fog; *rain* from 11½ A.M. |
| 21 | 29.698 | .123 | 63.0 | 3.2 | 61.0 | .536 | .84 | 0.013 | (Var.) | Cloudy; m. drizzling *rain*. |
| 22 | 29.731 | .059 | 56.7 | 6.3 | 43.5 | .283 | .54 | | W. | M. hazy, aft. cl'dy, ev. clear. |
| 23 | 29.810 | .079 | 47.0 | 9.7 | 40.0 | .247 | .71 | | (Var.) | Cloudy; aft. & night *rain*. |
| 24 | 29.579 | .231 | 46.5 | 2.8 | 45.8 | .308 | .80 | 1.304 | NNE. | Cl'y, *rain* continued all day |
| 25 | 29.826 | .247 | 41.7 | 4.8 | 26.9 | .146 | .47 | | W. | M. & ev. cl'r; aft. cl'y; *rain* mingled with *snow*. |
| 26 | 30.001 | .237 | 43.0 | 3.3 | 31.2 | .175 | .50 | | WSW. | M. & ev. cl'y; aft. cl'r. *Ther.* |
| 27 | 29.772 | .229 | 47.5 | 4.3 | 39.5 | .243 | .58 | 0.017 | (Var.) | Cl'dy; m. *rain*. [*lowest* 34° |
| 28 | 29.668 | .158 | 50.3 | 2.8 | 32.8 | .187 | .42 | | NW. | M. & aft. cloudy; ev. clear. |
| 29 | 30.052 | .384 | 48.2 | 3.5 | 34.3 | .198 | .43 | | SW. | M. hazy; aft. and ev. clear. |
| 30 | 29.927 | .125 | 57.7 | 9.5 | 53.6 | .412 | .60 | | SW. | M. cloudy; aft. & ev. clear. |
| 31 | 30.157 | .196 | 53.3 | 5.0 | 38.0 | .229 | .47 | | NE. | Cl'dy. *Bar. highest* 30.185. |
| Means for Oct., 1855 | 29.831 | .131 | 56.2 | 5.8 | 47.0 | .348 | .61 | 3.416 | S. 88½° W. | 70-100. |
| Means for Oct., 5 yrs. | 29.932 | .133 | 55.7 | 5.4 | 45.9 | | .61 | 2.763 | N. 73½° W. | 50-100. |

The Monthly Range of the Mercury in the Barometer was 0.732 of an inch, and in the Thermometer 43°.

